# Performance of computed tomography and magnetic resonance morphometry in evaluating brain atrophy in Down syndrome

**DOI:** 10.1002/alz.70296

**Published:** 2025-08-05

**Authors:** Beatriz Sánchez‐Moreno, Linda Zhang, Ana Carril Salaberry, Mateus Rozalem‐Aranha, Fernando Moldenhauer, Mikael Brudfors, John Ashburner, Parashkev Nachev, Diego Real de Asúa, Bryan A. Strange

**Affiliations:** ^1^ Adult Down Syndrome Unit, Department of Internal Medicine Hospital Universitario de La Princesa Universidad Autónoma de Madrid Madrid Spain; ^2^ Alzheimer Disease Research Unit, CIEN Foundation Queen Sofia Foundation Alzheimer Centre Madrid Spain; ^3^ Neuroradiology Section, Department of Radiology ‐ Hospital de la Santa Creu i Sant Pau Universitat Autònoma de Barcelona Barcelona Spain; ^4^ Memory Unit, Department of Neurology Institut de Recerca Sant Pau Hospital de la Santa Creu i Sant Pau Universitat Autònoma de Barcelona Barcelona Spain; ^5^ NVIDIA Corp London UK; ^6^ Wellcome Centre for Human Neuroimaging University College London London UK; ^7^ High‐Dimensional Neurology Group University College London Queen Square Institute of Neurology London UK; ^8^ Laboratory for Clinical Neuroscience, Centro de Tecnología Biomédica Universidad Politécnica de Madrid Madrid Spain

**Keywords:** Alzheimer's disease, computed tomography, Down syndrome, machine learning, magnetic resonance imaging, voxel‐based morphometry

## Abstract

**BACKGROUND:**

To compare the performance of computed tomography (CT) with that of magnetic resonance imaging (MRI) in assessing brain atrophy in Down syndrome (DS).

**METHODS:**

Retrospective comparison of CT‐derived gray and white matter measurements with those obtained by MRI using voxel‐based morphometry (VBM) in adults with DS who underwent both scanning modalities with a maximum interval of 1 year. The concordance of both tests was compared across sample quartiles.

**RESULTS:**

Eleven of 23 participants had some degree of cognitive decline. When comparing VBM outputs of both imaging techniques, an overlap of voxels negatively correlated with dementia severity was observed in the right temporal lobe. Observed agreement was 92.3% (quadratic‐weighted kappa index 0.71) for both gray and white matter.

**DISCUSSION:**

CT and MRI analyses showed a similar decrease in brain volume as cognitive decline increased. Our results highlight the potential use of CT in computational neuroanatomy studies of DS.

**Highlights:**

- Voxel‐based morphometry shows that computed tomography (CT) and magnetic resonance imaging (MRI) yield similar brain atrophy patterns in DS.
- Strong agreement (kappa index 0.71) was found between CT and MRI assessments.
- CT‐identified right temporal lobe volume loss linked to cognitive decline in DS.
- CT offers a practical, accessible alternative to MRI for brain imaging in DS.
- Artificial intelligence–based CT analysis enhances neuroimaging for DS with reduced motion issues.

## 1 BACKGROUND

Down syndrome (DS), resulting from the presence of an extra copy of chromosome 21,^1^ is considered a genetic form of Alzheimer's disease (AD),^2^ with an early onset of cognitive decline from the age of 40.^3^ The intersection of these two conditions poses unique challenges for accurate and timely diagnosis, particularly in people with severe or profound baseline intellectual disability. Although AD represents a major health problem for individuals with DS, conventional diagnostic techniques often face limitations in this specific population, where symptoms of dementia may be confused with the cognitive and functional diversity associated with DS.^4^

Among the various neuroimaging modalities used in the diagnosis and evaluation of neurodegenerative diseases, magnetic resonance imaging (MRI) is a valuable tool for assessing brain atrophy and other structural changes. MRI provides excellent soft tissue contrast and spatial resolution, enabling detailed visualization of brain structures. However, acquiring high‐quality MRI data in individuals with DS can be complex. Prolonged scan times can be poorly tolerated, leading to motion artifacts and the need for repeat scans, often requiring special considerations and even the use of sedation, with its additional risks.^5^ These limitations motivate the exploration of alternative imaging techniques that might offer accelerated acquisition without sacrificing diagnostic accuracy.

Computed tomography (CT), with its shorter acquisition times and more affordable costs, is commonly used in clinical practice and could represent a promising neuroimaging method in individuals with intellectual disabilities. Despite these practical advantages, the utility of CT in diagnostics, as well as volumetric approaches, has traditionally been hampered by its limited contrast resolution. However, recent advances in artificial intelligence and automated image analysis could change this scenario, potentially improving the diagnostic capabilities of CT.^6^ The development of new algorithms enables the extraction of additional valuable information from CT scans that may also help to understand the structural changes associated with neurodegenerative disorders.

In previous work^7^ we applied an automated algorithm—CTseg (https://github.com/WCHN/CTseg)—to brain CT images obtained for clinical reasons in individuals with DS. This algorithm was able to spatially normalize and segment CT images, allowing for detailed morphometric analysis.^8^ In the present study we sought to validate our former findings by comparing the performance of CTseg analysis with traditional brain MRI morphometry in the assessment of brain atrophy in a follow‐up cohort of adults with DS. Our hypothesis was that both imaging modalities would yield similar results in voxel‐based morphometric analysis, identifying the most significantly reduced brain areas according to the stage of dementia. Furthermore, we aimed to determine the concordance between the techniques in measuring total brain volumes, which would validate CT as a reliable alternative to MRI in this population.

## 2 METHODS

### 2.1 Study design, setting, and participants

This retrospective cross‐sectional study focused on individuals with DS, who were evaluated at a specialized adult outpatient clinic at a tertiary care university hospital in Madrid, Spain. We initially selected participants with a confirmed diagnosis of DS, either with a compatible karyotype or typical phenotype, who had undergone CT and MRI brain scans less than 1 year apart and who had consented to participate. These scans had been performed for clinical reasons, or due to prior participation in other research projects. Taking into account these criteria, the entire cohort available since the digitalization of medical records (from 2004 to 2023) was included, in order to maximize the potential recruitment of subjects.

### 2.2 Clinical variables

Clinical data were collected retrospectively from participants' medical records. Sociodemographic information included age, sex, estimated socioeconomic status (based on the median income level of the district of residence), educational level, and occupation (if applicable). Family history of AD or other dementias and common comorbidities in the DS population, such as congenital heart disease, obstructive sleep apnea, thyroid disorders, hearing loss, visual acuity loss, and epilepsy, were also documented. In addition, the use of medication affecting the central nervous system at the time of the scans was noted.

Baseline functional status was assessed using Part 1 of the Dementia Screening Questionnaire for People with Intellectual Disability (DSQIID).^9^ This section of the scale rates speech, daily living skills, and living location. For the purposes of our study, we assigned ordinal scores to each item, obtaining from their sum a total score that could range from 0 to 8. Higher scores indicated greater dependence on daily functioning, so this measure could be used to estimate participants' pre‐existing functional limitations prior to the onset of cognitive decline. Both the DSQIID score and the cognitive status of participants at the time of inclusion in the study were determined using information provided by caregivers and the clinical assessment contained in their medical records. Cognitive status was categorized into three groups, broadly following the recommendations of the American Association on Mental Retardation ‐ International Association for the Scientific Study of Intellectual Disabilities (AAMR‐IASSID) Working Group for the Establishment of Criteria for the Diagnosis of Dementia in Individuals with Developmental Disabilities: (1) cognitively stable (CS), in the absence of clinically significant cognitive impairment; (2) mild cognitive impairment (MCI), characterized by signs of cognitive impairment without accompanying functional impairment; and (c) established dementia, either moderate or severe, indicating a relevant loss of autonomy.^10^ Symptoms associated with cognitive impairment, such as seizures, depression, behavioral disturbances, and changes in sleep and/or gait, were also registered.

### 2.3 Imaging data acquisition and processing

CT scans were performed at La Princesa Hospital in Madrid between 2004 and 2023 for clinical purposes. These scans were acquired using either a 64‐channel Toshiba Aquilion system with a resolution of 0.4 × 0.4 × 3 mm^3^ or a 64‐channel Siemens Sensation system with a resolution of 0.4 × 0.4 × 2.4 mm^3^. MRI scans were obtained either for clinical purposes at La Princesa Hospital between 2004 and 2023 or as part of another research project at the CIEN Foundation in Madrid between 2021 and 2023. The MRI scans at La Princesa Hospital were acquired using a General Electric Explorer MRI, whereas the CIEN Foundation was equipped with a General Electric 3T MRI (Signa HDxt GEHC, Waukesha, USA). The parameters used were the following: repetition time 10 ms, echo time 4.5 ms, inversion time 600 ms, field of view 240 mm, matrix 288 × 288, and slice thickness 1 mm, resulting in a voxel resolution of 0.5 × 0.5 × 1 mm.

Prior to analysis, brain scans were anonymized, and processing was performed blind to clinical records. In cases where posterior fossa and supratentorial acquisitions were conducted separately, these images were fused to create a unified three‐dimensional image. An initial visual inspection of the studies was carried out by an investigator (B.S.M.), and scans with missing T1 sequences, significant motion artifacts, or faulty merging were excluded from the analysis.

**RESEARCH IN CONTEXT**

**1. Systematic review**: A thorough review of the literature on PubMed shows that comparisons of the performance of brain computed tomography (CT) versus magnetic resonance imaging (MRI) to evaluate cortical atrophy are scarce, and precede the systematic use of artificial intelligence processing. Our previous experience with a CT automated algorithm in Down syndrome (DS) led us to hypothesize that it could be as useful as MRI for this purpose.

**2. Interpretation**: Our results showed that CT and MRI behaved similarly in detecting a decrease in brain volume as cognitive impairment progresses in people with DS. These findings are consistent with other studies and with our usual clinical practice and could contribute to a more accessible and cost‐effective diagnosis of AD in this population.

**3. Future directions**: This study sets the stage for longitudinal studies investigating imaging test performance in detecting regional brain atrophy over time. We also found a lateralization in the pattern of brain atrophy, which would need further clarification in future studies.

CT scans were then processed with CTseg (Wellcome Trust Centre for Neuroimaging, University College London; https://github.com/WCHN/CTseg), which uses flexible Bayesian modeling to spatially normalize images to standard Montreal Neurological Institute (MNI) space and segment them into standard tissue classes, including gray matter (GM), white matter (WM), and cerebrospinal fluid (CSF).^8^ After segmentation, images were smoothed with a Gaussian kernel of 6 mm full width at half maximum. Total intracranial volume (TIV) was calculated by summing the total GM, WM, and CSF values for each participant. This value was used to adjust for individual differences in head size in all volume‐based analyses.

For MRI images, structural pre‐processing of T1 sequences was performed using Statistical Parametric Mapping (SPM). Before segmentation, the skull was stripped using MRIcroGL (FMRIB Image Analysis Group, Oxford, UK). SPM DARTEL algorithms were implemented for segmentation into GM, WM, and CSF, and, subsequently, for the generation of spatially normalized, modulated, and smoothed partitions (8 × 8 × 8 mm full‐width at half‐maximum). TIV was calculated in a similar manner as for CT scans.

The automatically segmented CT and MRI results were also visually inspected by two authors (B.S.M. and L.Z.) to ensure accuracy, and those scans with significant segmentation errors were likewise excluded from the analysis.

### 2.4 Ethical considerations

This study was conducted in compliance with the principles outlined in the Declaration of Helsinki^11^ and was approved by the local institutional review board with registration no. 3911/2019 (Instituto de Investigación Sanitaria del Hospital de La Princesa). Data confidentiality was guaranteed as regulated by current Spanish legislation.^12^

### 2.5 Statistical analysis and voxel‐based morphometry (VBM) analysis

Stata software (Stata v15.0) was used for clinical data analysis. All statistical tests were two tailed, with a significance level set at *p* < .05. Correction for multiple comparisons was implemented using Bonferroni or Tukey methods, where applicable. To evaluate potential selection bias, baseline clinical characteristics were compared between initially included and finally selected individuals, excluding those with poor image quality that precluded VBM analyses, as discussed above.

VBM analyses were carried out using SPM12 (http://www.fil.ion.ucl.ac.uk/spm/). Smoothed and modulated GM and WM images were separately correlated with the degree of cognitive decline/dementia severity using two different general linear models (GLMs) for the CT and MRI datasets. This choice was made to avoid systematic biases inherent in direct comparison of MRI and CT at the voxel level, given the fundamental differences in how each modality captures tissue properties. Age, gender, and TIV were treated as potential confounders, based on previous findings,^7^ and included as nuisance regressors. All results were corrected for multiple comparisons using family‐wise error (FWE) correction. In addition, within each imaging modality, we planned to investigate the influence of the specific scanner type by incorporating it as an additional covariate in the analysis.

Parallel to the quantitative analysis, a qualitative assessment was carried out to detect differences by brain area, which the overall quantitative analysis alone might not have captured.

Furthermore, to explore the relationship between brain volumes obtained from both imaging modalities, we performed a Pearson correlation analysis between GM, WM, and total brain volume (TBV) estimates from CT and MRI. Given the inherent differences in image resolution and normalization parameters between CT and MRI, direct voxel‐wise quantitative comparisons were not feasible. Therefore, to further assess concordance between CT and MRI findings, a kappa index analysis was conducted. The study population was divided into quartiles based on GM and WM total density, and a quadratic‐weighted kappa index, suitable for ordinal data analysis, was applied to evaluate the level of agreement between CT and MRI assessments.

## 3 RESULTS

### 3.1 Clinical characteristics of the study cohort

**FIGURE 1:**
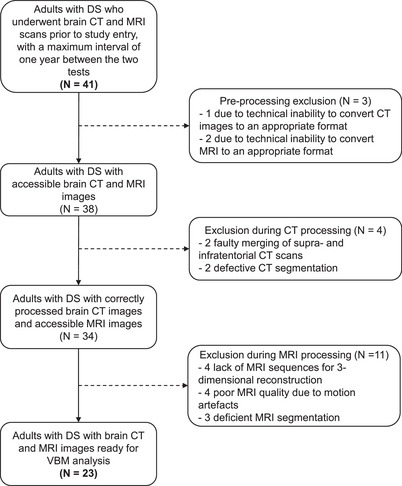
Flow diagram detailing the inclusion/exclusion of individuals in the study. CT, computed tomography; DS, Down syndrome; MRI, magnetic resonance imaging; VBM, voxel‐based morphometry.

The initial study cohort comprised 41 individuals but, as shown in Figure 1, after excluding cases with inaccessible brain CT or MR images, motion artifacts, or poor pre‐processing, the final study sample consisted of 23 participants. Their mean age was 52.4 years, ranging from 44.1 to 62.1 years, and 52.2% were women. Among them, 11 (47.8%) had cognitive impairment, of whom 7 were diagnosed with MCI and 4 had dementia. No relevant demographic or clinical differences were observed between the initial and final cohorts of the study, as shown in Table 1. All MRI scans included in this analysis were performed between 2021 and 2023 at the CIEN Foundation, except for one which was performed in 2014 at La Princesa Hospital. The median interval between CT and MRI was 121 days, and was inferior to 6 months in 18 cases (78.3%); in the remaining five participants, the interval ranged from 6 to 12 months, well within the range established in the study's inclusion criteria.

**TABLE 1:** Clinical characteristics of individuals initially included in the study versus those finally selected for imaging analysis.

Variables	Initial sample (*n* = 41)	Included participants (*n* = 23)	*p*‐value^a^
Median age in years (IQR)	50.6 (7.85)	52.3 (5.09)	0.010
Female sex	20 (48.8%)	12 (52.2%)	0.623
Family history of dementia	3 (7.32%)	3 (13.0%)	0.243
Income level			
High	15 (36.6%)	7 (30.4%)	0.579
Low	14 (34.2%)	8 (34.8%)	
Education			
None	1 (2.44%)	0	0.598
Special	23 (56.1%)	15 (65.2%)	
Inclusive	5 (12.2%)	3 (13.0%)	
Occupation			
None	12 (29.3%)	6 (26.1%)	1.000
Centre	27 (65.9%)	15 (65.2%)	
Integration	1 (2.44%)	1 (4.35%)	
Living situation			
Family home	29 (70.7%)	16 (69.6%)	0.759
Group home	3 (7.32%)	1 (4.35%)	
Residence	8 (19.5%)	5 (21.7%)	
DSQIID median score^b^ (IQR)	1 (1)	1 (0)	0.001
Congenital heart disease	5 (12.2%)	2 (8.70%)	0.297
Suspected or definite sleep apnea	13 (31.7%)	5 (21.7%)	0.301
Hypothyroidism	27 (65.9%)	16 (69.6%)	0.571
Visual impairment	25 (61.0%)	15 (65.2%)	0.529
Hearing impairment	7 (17.1%)	6 (26.1%)	0.112
Gait instability	8 (19.5%)	3 (13.1%)	0.710
Depression	9 (22.0%)	4 (17.4%)	0.471
Behavioral changes	9 (22.0%)	3 (13.0%)	0.147
Sleep cycle disorder	4 (9.76%)	0	0.030
Use of neuroleptic drugs	13 (31.7%)	8 (34.8%)	0.456
Use of benzodiazepines	3 (7.32%)	2 (8.70%)	1.000
Antiepileptic treatment	8 (19.5%)	2 (8.70%)	0.097
Cognitive state			
Stable	24 (58.5%)	12 (52.2%)	0.491
MCI	12 (29.3%)	7 (30.4%)	
Dementia	5 (12.2%)	4 (17.4%)	

Abbreviations: DSQIID, Dementia Screening Questionnaire for Individuals with Intellectual Disabilities; IQR, interquartile range; MCI, mild cognitive impairment.

^a^ The *p*‐values are the result of the comparison between included and excluded individuals, using the Wilcoxon test for quantitative variables and the chi‐square test for qualitative variables (Fisher's correction when necessary).

^b^ DSQIID Part 1 score ranges from 0 to 8, with higher scores indicating greater baseline dependence in daily living skills and communication.

### 3.2 Correlation between whole‐brain VBM analysis and clinical stage of AD

Mean estimates of GM volume and WM volume as a function of AD stage, obtained by both brain CT and MRI, are listed in Table 2. When adjusting for confounding variables (age, sex, and TIV), a negative correlation between volume and degree of dementia was observed in all multivariate linear regressions. This correlation was statistically significant (*p* < .05) for GM and WM on both CT and MRI, as can be seen in Table 3. The addition of other covariates (such as the degree of intellectual disability or behavioral disorders) did not appreciably modify the significance of these results.

**TABLE 2:** Association between brain volumes (GM, WM, and TBV) and the degree of AD with different imaging modalities.

(a) CT						
Brain volumes	CS	MCI	Dementia	Unadjusted F	Adjusted F	Adjusted *p*
GM	488.8	521.0	490.6	F(2,20) = 0.85	F(4,18) = 3.17	0.04
GM/TIV	0.46	0.45	0.44			
WM	370.1	391.3	359.6	F(2,20) = 0.70	F(4,18) = 3.71	0.02
WM/TIV	0.35	0.34	0.32			
TBV	859.0	912.3	850.2	F(2,20) = 0.77	F(4,18) = 4.11	0.02
TBV/TIV	0.81	0.80	0.77			

(b) MRI						
Brain volumes	CS	MCI	Dementia	Unadjusted F	Adjusted F	Adjusted *p*
GM	555.2	558.1	474.4	F(2,20) = 2.70	F(4,18) = 4.41	0.01
GM/TIV	0.47	0.45	0.35			
WM	345.6	338.1	342.9	F(2,20) = 0.06	F(4,18) = 4.96	0.01
WM/TIV	0.29	0.27	0.25			
TBV	900.8	896.2	817.3	F(2,20) = 1.03	F(4,18) = 4.88	0.01
TBV/TIV	0.76	0.71	0.61			

*Note*: Adjusted analysis included age, sex, and total intracranial volume as covariates.

Abbreviations: CS, cognitively stable; CT, computed tomography; GM, gray matter; MCI, mild cognitive impairment; MRI, magnetic resonance imaging; TBV, total brain volume; TIV, total intracranial volume; WM, white matter.

**TABLE 3:** Regression coefficients (b) and 95% confidence intervals from linear regression models predicting dementia status as a function of brain volume and covariates.

Image modality	Volumes	Coefficient (b)	95% CI	F(4,18)	*p*
CT	GM	−0.02	−0.04 to 0.001	3.17	0.039
	WM	−0.02	−0.04 to −0.003	3.71	0.023
MRI	GM	−0.004	−0.01 to 0.0005	4.41	0.011
	WM	−0.01	−0.02 to −0.0004	4.96	0.007

*Note*: Separate models were conducted for CT and MRI. Positive coefficients indicate a greater likelihood of dementia with increasing values of the predictor, whereas negative coefficients imply an inverse relationship. Models were adjusted for age, sex, and total intracranial volume.

Abbreviations: CI, confidence interval; CT, computed tomography; GM, gray matter; MRI, magnetic resonance imaging; WM, white matter.

**FIGURE 2:**
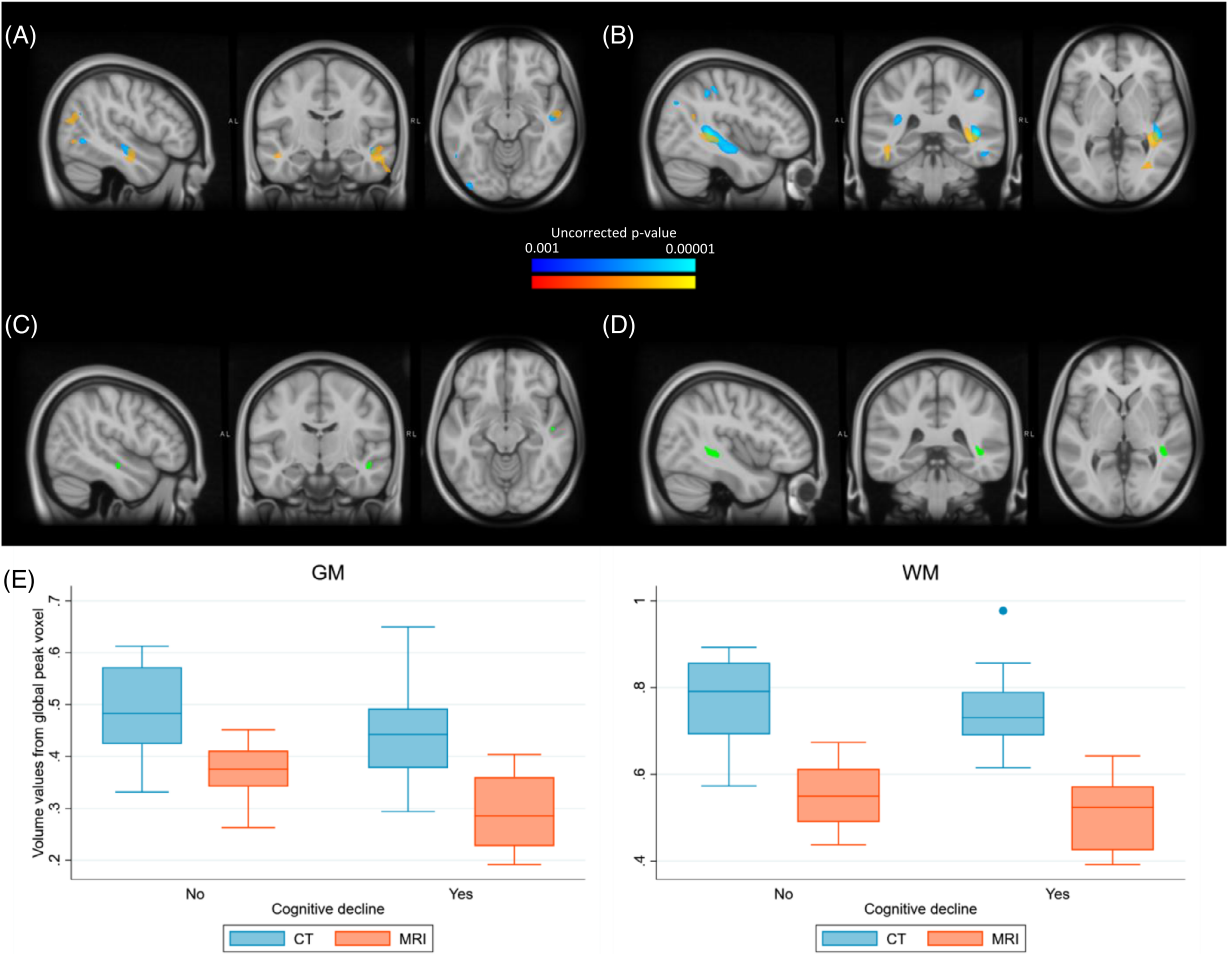
VBM analysis of gray and white matter from brain CT and MRI scans. (A) and (B) display the group‐level VBM correlations with cognitive status for GM and WM, respectively, overlaid on an average MRI template for visualization. Blue areas represent clusters of voxels from CT scans showing a negative correlation with AD severity, whereas orange areas reflect the same for MRI scans. The green areas in (C) and (D) indicate the regions where there is significant overlap between the two imaging techniques for GM and WM, respectively. (E) Shows the distribution of the volume values from the global peak voxel (MNI: 48, −13, −12 for GM and 42, −38, 1 for WM) as a function of the presence or absence of cognitive impairment, using box plots. A decreasing trend in these values is observed for both imaging modalities. CT, computed tomography; GM, gray matter; MRI, magnetic resonance imaging; WM, white matter; VBM, voxel‐based morphometry.

Regarding voxel‐wise, whole‐brain morphometric analysis, for both GM and WM, we tested for the contrast with AD stage for each imaging modality separately. When comparing clusters of voxels with a significant negative correlation (*p* < .001, uncorrected) with the degree of AD, after adjusting for the aforementioned confounding variables, an overlap between both imaging techniques was observed in the right temporal lobe (medial temporal gyrus for GM, inferior longitudinal fasciculus for WM), as can be observed in Figure 2. No statistically significant associations were found after applying FWE correction.

### 3.3 Concordance between brain CT and MRI measurements

**FIGURE 3:**
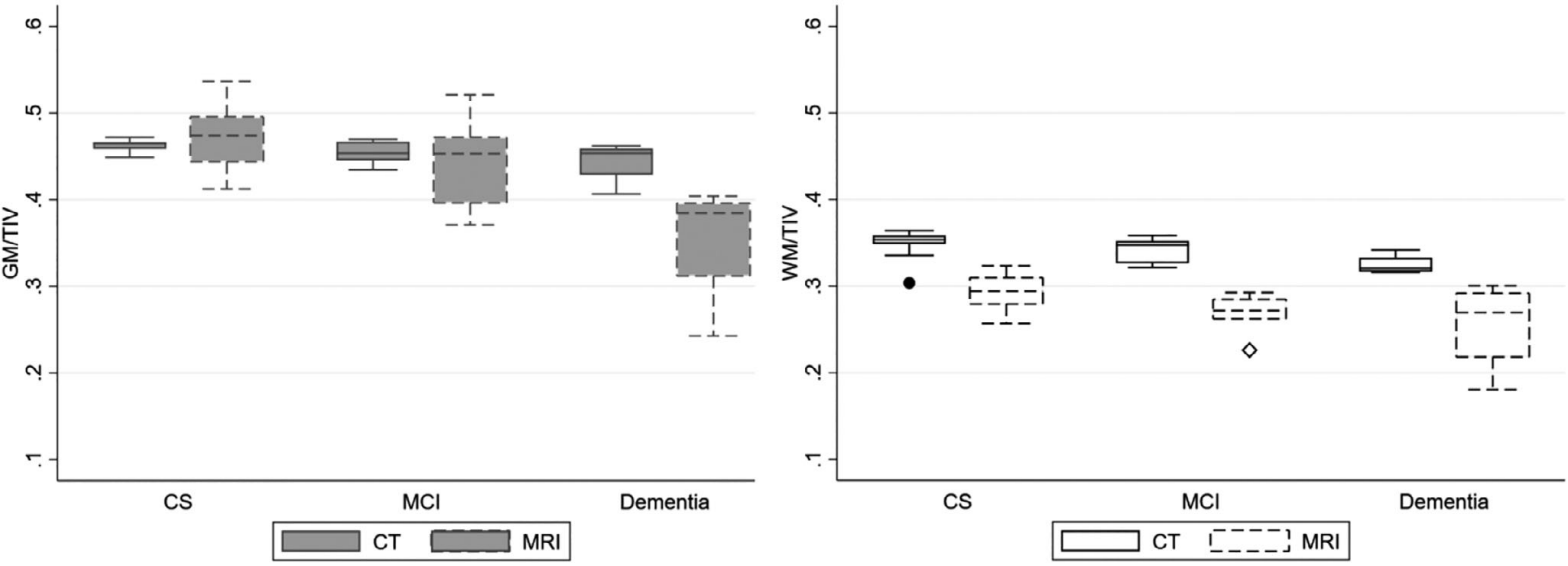
Box plots of TIV‐adjusted GM and WM, disaggregated by imaging technique and degree of AD. The values in the Y axis represent the global volume of GM and WM for each imaging modality, adjusted for TIV. CS, cognitively stable; CT, computed tomography; GM, gray matter; MCI, mild cognitive impairment; MRI, magnetic resonance imaging; TIV, total intracranial volume; WM, white matter.

We identified a statistically significant correlation between the volumes estimated by brain CT and MRI, despite the utilization of distinct algorithms (GM: *r* = 0.73; WM: *r* = 0.75; TBV: *r* = 0.82; *p* < .001 for all correlations). Upon examination of the total volumes adjusted for TIV, as illustrated in detail in Figure 3, an overlap was noted in the GM volumes measured with CT and MRI, especially in the CS and MCI groups. However, in the case of WM, CT exhibited a tendency toward higher WM/TIV volumes compared to MRI.

**FIGURE 4:**
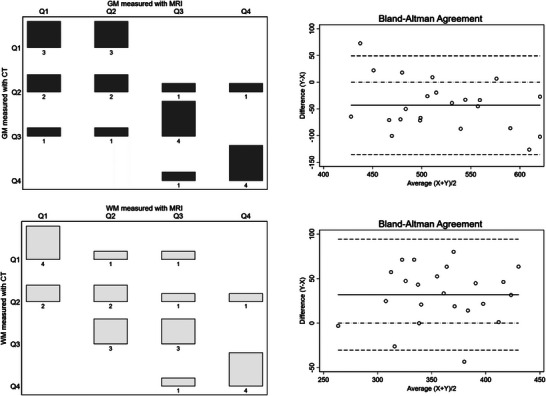
Division of the study sample into quartiles according to GM and WM, indicating the frequency of concordance in the classification obtained with CT and MRI. The corresponding Bland–Altman plot (for GM and WM, respectively) is shown next to each graph. In these plots, the solid horizontal line represents the mean difference (bias) between CT and MRI estimates. The dash‐dotted line at zero represents perfect agreement, whereas the additional dashed lines indicate the limits of agreement (±1.96 SD from the mean difference). GM, gray matter; CT, computed tomography; MRI, magnetic resonance imaging; Q, quartile; WM, white matter.

After dividing the study sample into quartiles of GM, WM, and TBV measured by brain CT and MRI, concordance analysis using quadratic‐weighted kappa demonstrated high agreement between the two imaging techniques. The observed agreement ranged from 92.3% for GM and WM to 95.2% for TBV, with corresponding quadratic‐weighted kappa values from 0.7107 to 0.8192. These results indicate substantial agreement beyond what would be expected by chance alone, suggesting strong concordance between CT and MRI measurements across the quartiles. Figure 4 shows how CT and MRI rank the sample by quartiles for both GM and WM. In the Bland–Altman plots, only one case (4.35% of the total sample) is found above the concordance limit for GM and one below the limit for WM.

## 4 DISCUSSION

Our findings reveal a comparable performance between CT and MRI in estimating brain volumes through VBM in adults with DS. First, despite the differences in imaging methodologies, VBM analysis found GM and WM volume reductions in overlapping regions of the temporal lobes as AD progresses, particularly on the right side, encompassing the medial temporal gyrus for GM and the inferior longitudinal fasciculus for WM. Although the hippocampus, a key structure in AD pathology, could not be reliably assessed with CT‐based VBM, the involvement of adjacent regions may indirectly reflect hippocampal and parahippocampal changes. This observation aligns with our previous study, which analyzed CT‐derived images in a larger DS population and similarly noted a right lateralized WM volume loss extending toward the inferior longitudinal fasciculus with worsening cognitive status.^7^ Moreover, our prior dysconnectome analysis also showed that GM volume loss in the right lateral temporal cortex could be attributed to WM integrity loss in that area. In another longitudinal MRI study in individuals with DS, both GM and WM reductions were also found in the right orbitofrontal cortex and right temporal lobe.^13^ In contrast, another recent study, which measured GM using MRI in adults with DS, also identified lateralized differences based on cued recall test scores and plasma neurofilament light chain levels; however, in this publication, the atrophy was more prominent on the left side, affecting anterolateral entorhinal cortex thickness.^14^

Other studies in the general population have described asymmetries in relation to AD, typically showing predominantly left‐sided atrophy.^15^, ^16^, ^17^, ^18^ This observation is consistent with that of another study, also conducted in AD patients without DS, in which MRI‐ and CT‐derived VBM were compared.^19^ The latter was able to find more extensive areas of atrophy (right caudate head, left anterior cingulate, and right temporal pole), leading Imabayashi et al. to hypothesize that CT might be more sensitive and even suggest that it could replace MRI. The cause of this asymmetry in AD neurodegeneration is still unknown.^20^ Although data on hand dominance could have provided more insight into this issue, this information was not available in our study or in the other referenced studies. In summary, lateralization in the pattern of atrophy was an incidental finding, and its significance remains unclear; nonetheless, its explanation is beyond the scope of this research and should be clarified in further studies.

Second, our study provides evidence of concordance between computational neuroanatomic measures derived from brain CT and MRI. Specifically, we observed high correlations for GM, WM, and TBV measurements, and quadratic‐weighted kappa values suggested strong agreement between the two imaging modalities. However, GM density appears to be consistently higher on MRI than on CT, whereas the opposite is true for WM. This could be explained by the fact that CT and MRI capture different tissue contrasts due to inherent discrepancies in image resolution and tissue composition. MRI provides superior soft tissue differentiation, which could lead to a more accurate segmentation of GM. In contrast, CT scans are highly sensitive to differences in tissue density. Despite these dissimilarities, our study shows a similar performance of both tests in detecting brain atrophy as a function of AD degree.

These results support the utility of brain CT as a viable alternative to MRI, which may be less accessible or tolerable for people with DS, as seen by the high exclusion rate due to problems with MRI. Most exclusions were due to poor image quality, highlighting the difficulty of obtaining reliable MRI scans in this population. This mirrors previous studies in which 12%–36% of the participants had to be withdrawn for similar reasons.^13^, ^21^, ^22^, ^23^ Thus, CT offers a practical alternative for assessing brain structure in individuals with DS with faster acquisition times and better tolerability.

To the best of our knowledge, this is the first study in adults with DS to compare brain CT and MRI on a person‐to‐person basis. A major strength of this study is that the images were obtained primarily from routine clinical practice, allowing for the extrapolation of our findings to real‐world settings. In addition, we successfully extracted further information from the scans with advanced automated analysis technology, employing free, publicly accessible artificial intelligence tools.

Despite these strengths, several limitations should be acknowledged. As a cross‐sectional study, it is not possible to determine progressive brain changes in each participant. The inter‐scan interval, of up to 1 year, might have introduced some variability between brain CT and MRI estimates, especially in the context of rapidly progressive AD associated with DS; nonetheless, given that visible neurodegenerative changes in AD progress gradually over years rather than months,^24^, ^25^ and that most scans were performed within a short period, it is unlikely that this factor significantly influenced our findings.

The retrospective and observational nature of the study also limits the reliability of the data collected. Some clinical variables prone to subjective interpretation, such as the classification of AD stage, may have been subject to bias. The quality of the images may have been affected by the use of different types of scanners over various time periods. Even so, in the final sample, all analyzed images were acquired in recent years on the same devices, except for one participant. This suggests that older scans were more likely to be excluded due to quality limitations, ultimately improving the homogeneity of the final dataset. In addition, preprocessing and normalization steps likely helped minimize systematic differences between scanner models.

The small sample size, particularly in the dementia subgroup, may limit the generalizability of our findings and reduce the power of the study to detect statistically significant differences; in fact, the statistical significance of the correlation between decreased brain volume and degree of dementia on both CT and MRI does not hold after correction for multiple comparisons in the VBM analysis. However, our results are aligned with previous studies with larger sample sizes, in which the relationship between regional brain atrophy and dementia has already been demonstrated.^7^ Furthermore, our current hypothesis was that both imaging tests are comparable. It is worth mentioning that technical exclusions affected MRI more than CT (13 vs 5 cases), reinforcing the potential practicality of CT in clinical settings and supporting its broader applicability in individuals with DS. Given these factors, our findings should be interpreted as exploratory, providing a foundation for future studies with larger cohorts. Furthermore, the high number of exclusions could have led to selection bias, as individuals who completed the study likely have a lower degree of intellectual disability (as indicated by differences in the DSQIID scale), as well as fewer behavioral disorders and less epilepsy. Although an effort was made to include a diverse sample with different levels of cognitive ability, the underrepresentation of these participants, likely due to poor tolerance to the scans, may compromise the external validity of the study and should be taken into account when interpreting the results.

Finally, when considering the applicability of CT, it is important to note that, although it is used widely in clinical practice, it carries inherent risks due to radiation exposure, even though the global risk of developing solid tumors is negligible in the DS population.^26^, ^27^ The use of specialized image processing software, which requires technical expertise and is not yet widely available in routine clinical practice, may also limit its broader applicability. However, this study was designed as a proof‐of‐concept, aiming to explore the utility of brain CT as a complementary tool for the diagnosis of AD in DS. Future advancements in user‐friendly software interfaces, automated pipelines, or integrated clinical platforms could help overcome this limitation and facilitate wider adoption of this type of analysis.

In conclusion, our results suggest that brain CT provides reliable morphometric data comparable to MRI in adults with DS, making it a valuable tool for evaluating AD‐associated cognitive decline in individuals with DS. This could facilitate more widespread brain imaging, aiding early detection and monitoring of AD progression. Future research should explore the pathophysiological relationship between regional brain changes detected by VBM and cognitive decline. Longitudinal studies are needed to investigate the progression of neuroanatomic changes over time. Furthermore, the overall volume reductions observed in association with dementia severity raise the question of whether specific thresholds could be established to support clinical interpretation in individual cases, which would significantly facilitate the implementation of our findings in clinical practice.

## AUTHOR CONTRIBUTIONS

Design: Beatriz Sánchez‐Moreno and Diego Real de Asúa. Data collection: Beatriz Sánchez‐Moreno, Ana Carril Salaberry, Fernando Moldenhauer, and Diego Real de Asúa. Data analysis and interpretation: Linda Zhang, Beatriz Sánchez‐Moreno, Diego Real de Asúa, Mikael Brudfors, John Ashburner, and Parashkev Nachev. Manuscript drafting and revision: Linda Zhang, Beatriz Sánchez‐Moreno, Diego Real de Asúa, and Mateus Rozalem‐Aranha. Approval of the final version: All.

## CONFLICT OF INTEREST STATEMENT

Mateus Rozalem‐Aranha has provided paid consultancy for Veranex. Mateus Rozalem‐Aranha is a partner and director of production at Masima‐Soluções em Imagens Médicas LTDA. All other authors (Beatriz Sánchez‐Moreno, Linda Zhang, Ana Carril Salaberry, Fernando Moldenhauer, Mikael Brudfors, John Ashburner, Parashkev Nachev, Diego Real de Asúa, and Beatriz Sánchez‐Moreno) have no conflicts of interest to disclose. Author disclosures are available in the Supporting Information.

## CONSENT STATEMENT

The institutional review board approved the exemption from seeking informed consent due to the absence of intervention in the study population and the retrospective and anonymous nature of the study.

## Supporting information




Supporting Information

Supporting Information

Supporting Information
