# Supplementary material for: Performance of computed tomography and magnetic resonance morphometry in evaluating brain atrophy in Down syndrome
Source: Alzheimers Dement. 2025 Aug 5;21(8):e70296. doi: 10.1002/alz.70296 (PMC12322686; doi:10.1002/alz.70296)
Supplement: Supplementary file 2 — Supporting Information [file ALZ-21-e70296-s003.docx]

| ICMJE DISCLOSURE FORM | |
| --- | --- |
| **Date:** | March 11th, 2025 |
| **Your Name:** | Diego Real de Asúa Cruzat |
| **Manuscript Title:** | Performance of computed tomography and magnetic resonance morphometry in evaluating brain atrophy in Down syndrome |
| **Manuscript Number (if known):** | ADJ-D-24-02725 |
| In the interest of transparency, we ask you to disclose all relationships/activities/interests listed below that are related to the content of your manuscript. “Related” means any relation with for-profit or not-for-profit third parties whose interests may be affected by the content of the manuscript. Disclosure represents a commitment to transparency and does not necessarily indicate a bias. If you are in doubt about whether to list a relationship/activity/interest, it is preferable that you do so.  The author’s relationships/activities/interests should be defined broadly. For example, if your manuscript pertains to the epidemiology of hypertension, you should declare all relationships with manufacturers of antihypertensive medication, even if that medication is not mentioned in the manuscript.  In item #1 below, report all support for the work reported in this manuscript without time limit. For all other items, the time frame for disclosure is the past 36 months. | |

|  | | | **Name all entities with whom you have this relationship or indicate none (add rows as needed)** | **Specifications/Comments (e.g., if payments were made to you or to your institution)** |
| --- | --- | --- | --- | --- |
| **Time frame: Since the initial planning of the work** | | | | |
| **1** | All support for the present manuscript (e.g., funding, provision of study materials, medical writing, article processing charges, etc.)  **No time limit for this item.** | | Article processing charges will be supported through the corresponding author’s institution (Universidad Autónoma de Madrid) | |
| **Time frame: past 36 months** | | | | |
| **2** | | Grants or contracts from any entity (if not indicated in item #1 above). | 1. Randomized controlled trial: Ensayo en fase III para evaluar la seguridad y eficacia de LEvetiracetam para prevenir las crisiS epilépticas en adultos con síndrome de Down y enfermedad de Alzheimer. (Funded by Instituto de Salud Carlos III, ref. No. ICI23/32; dates 01/01/2024-31/12/2028).  2 Observational research project: Analysis of the immune response before and after vaccination against SARS-CoV-2 in adults with Down syndrome. (Funded by Fondation Jérôme Lejeune, ref. No. 2021a/2069; 2021-2023)  3 Observational research project: Estudio de los mecanismos protectores frente al desarrollo de hipertensión arterial en adultos con Síndrome de Down. (Funded by Instituto de Salud Carlos III), ref. no. PI19/00634; 01/01/2020-31/12/2022) | |
| **3** | | Royalties or licenses | **None** | |
| **4** | | Consulting fees | **None** | |
| **5** | | Payment or honoraria for lectures, presentations, speakers bureaus, manuscript writing or educational events | UNIVERSIDAD COMPLUTENSE DE MADRID: Master “Paciente con enfermedad crónica avanzada y necesidades paliativas” (ongoing)  WEILL CORNELL MEDICINE-QATAR: Division of Medical Ethics – several courses (ongoing)  INSTITUTO UNIVERSITARIO DE INVESTIGACIÓN ORTEGA Y GASSET: Máster oficial en bioética clínica (ongoing)  Servicio Aragonés de Salud: Curso FORMACIÓN EN CONSULTORÍA DE ÉTICA CLÍNICA (2022)  ESCUELA ANDALUZA DE SALUD PÚBLICA: Curso Consultoría Ética Clínica (2022) y Máster en bioética (2022)  CONSEJERÍA DE SALUD, COMUNIDAD DE MADRID: Curso Consultoría Ética Clínica (nov 2021) | |
| **6** | | Payment for expert testimony | **None** | |
| **7** | | Support for attending meetings and/or travel | **None** | |
| **8** | | Patents planned, issued or pending | **None** | |
| **9** | | Participation on a Data Safety Monitoring Board or Advisory Board | **None** | |
| **10** | | Leadership or fiduciary role in other board, society, committee or advocacy group, paid or unpaid | **None** | |
| **11** | | Stock or stock options | **None** | |
| **12** | | Receipt of equipment, materials, drugs, medical writing, gifts or other services | **None** | |
| **13** | | Other financial or non-financial interests | **None** | |
|  | |  |  | |
| **Please place an “X” next to the following statement to indicate your agreement:** | | | | |
| X | | I certify that I have answered every question and have not altered the wording of any of the questions on this form. | | |
